# The Invertebrate-Derived Antimicrobial Peptide Cm-p5 Induces Cell Death and ROS Production in Melanoma Cells

**DOI:** 10.3390/md23070273

**Published:** 2025-06-29

**Authors:** Ernesto M. Martell-Huguet, Daniel Alpízar-Pedraza, Armando Rodriguez, Marc Zumwinkel, Mark Grieshober, Fidel Morales-Vicente, Ann-Kathrin Kissmann, Markus Krämer, Steffen Stenger, Octavio L. Franco, Ludger Ständker, Anselmo J. Otero-Gonzalez, Frank Rosenau

**Affiliations:** 1 Center for Protein Studies, Faculty of Biology, University of Havana, 25 and I, La Habana 10400, Cuba; nestmartell@gmail.com; 2 Core Facility for Functional Peptidomics, Ulm Peptide Pharmaceuticals (U-PEP), Faculty of Medicine, Ulm University, 89081 Ulm, Germany; armando.rodriguez-alfonso@uni-ulm.de (A.R.); ludger.staendker@uni-ulm.de (L.S.); 3 Center for Pharmaceutical Research and Development, 26th Avenue, No 1605, Nuevo Vedado, La Habana 10400, Cuba; dalpizarp@gmail.com; 4 Core Unit of Mass Spectrometry and Proteomics, Faculty of Medicine, Ulm University, Albert-Einstein-Allee 11, 89081 Ulm, Germany; 5 Institute of Medical Microbiology and Hygiene, University Clinic of Ulm, Albert-Einstein-Allee 11, 89081 Ulm, Germany; marc.zumwinkel@uniklinik-ulm.de (M.Z.); mark.grieshober@uniklinik-ulm.de (M.G.); steffen.stenger@uniklinik-ulm.de (S.S.); 6 Synthetic Peptides Group, Center for Genetic Engineering and Biotechnology, La Habana 10600, Cuba; femvicente@gmail.com; 7 Institute of Pharmaceutical Biotechnology, Ulm University, Albert-Einstein-Allee 11, 89081 Ulm, Germany; ann-kathrin.kissmann@uni-ulm.de (A.-K.K.); markus-1.kraemer@uni-ulm.de (M.K.); 8 Center for Biochemical and Proteomics Analyses, Catholic University of Brasilia, Brasilia 71966-700, Brazil; ocfranco@gmail.com; 9 S-inova Biotech, Programa de Pós-Graduação em Biotecnologia, Universidade Católica, Dom Bosco Avenida Tamandaré 6000, Campo Grande 79117-900, Brazil

**Keywords:** Cm-p5, anti-cancer peptide, membrane disruption, cell death, ROS

## Abstract

Nowadays, healthcare systems face two global challenges: the rise of multidrug-resistant pathogens and the growing incidence of cancer. Due to their broad spectrum of activities, antimicrobial peptides emerged as potential alternatives against both threats. Our group previously described the antifungal activity of the α-helical peptide Cm-p5, a derivative of the natural peptide Cm-p1, isolated from the coastal mollusk *Cenchritis muricatus*; however, its anti-cancer properties remained unexplored. Analyses through calorimetry and molecular dynamics simulations suggest the relevance of phosphatidylserine for the attachment of Cm-p5 to cancer cell membranes. Cm-p5 exhibited cytotoxic activity in a dose-dependent manner against A375 melanoma cells, without toxicity against non-malignant cells or hemolytic activity. DAPI/PI and DiSC3(5) staining confirmed permeabilization, disruption, and depolarization of A375 cytoplasmic membranes by Cm-p5. Furthermore, Annexin V-FITC/PI assay revealed the induction of cellular death in melanoma cells, which can result from the cumulative membrane damage and oxidative stress due to the overproduction of reactive oxygen species (ROS). Moreover, after the treatment, the proliferation of A375 cells was dampened for several days, suggesting that Cm-p5 might inhibit the recurrence of melanomas. These findings highlight the multifunctional nature of Cm-p5 and its potential for treating malignant melanoma.

## 1. Introduction

The concurrent escalation of antimicrobial resistance (AMR) and cancer incidence represents a formidable challenge to global health [1]. In 2021, AMR infections were directly responsible for approximately 1.14 million deaths worldwide and contributed to 4.71 million deaths, underscoring their critical threat to public health [2]. The misuse and overuse of antibiotics in humans, animals, and plants are primary drivers in the development of drug-resistant pathogens, limiting treatment options for infectious diseases [3]. Concurrently, cancer remains a leading cause of mortality, with the World Health Organization reporting a significant increase in global cancer cases over recent decades, with an estimated 20 million new cancer cases and 9.7 million deaths in 2022 alone [4]. Conventional anti-cancer treatments have critical limitations because, in most cases, they generate resistance or several side effects [5]. Resistance arises from genetic and physiological variations of cancer cells in response to the recurrent use of chemotherapeutic drugs or due to changes in the tumor microenvironment [6,7]. In addition, tumor heterogeneity and the dynamics between different treatments, together with the particular response of each patient, contribute to further reducing the effectiveness of conventional therapies [8,9]. These two health crises, while distinct, intersect notably in clinical settings [10,11]. Patients undergoing cancer treatments, such as chemotherapy, often experience immunosuppression, rendering them more susceptible to infections [10]. The growing prevalence of AMR exacerbates this vulnerability, making infections more challenging to treat and increasing morbidity and mortality among cancer patients [11]. Notably, AMR rates for specific key pathogens were found to be 1.5 to 2 times greater in hospitalized cancer patients compared to non-cancer patients, highlighting the compounded risk in this population [11,12].

In response to these intertwined challenges, Host Defense Peptides (HDPs) and their synthetic derivatives have garnered attention in the field of biomedicine as a novel alternative to counteract tumors and multidrug-resistant pathogens [13,14]. HDPs, also called antimicrobial peptides (AMPs), are small peptides (5 to 50 aminoacids) that generally contain high proportions of cationic and hydrophobic residues, which give them a positive net charge at physiological pH and the capacity to fold into α-helices, β-sheets, extended helices, loops, and turns [15]. Initially recognized for their role in innate immunity against pathogens, certain HDPs have demonstrated selective cytotoxicity against cancer cells, classifying them as anti-cancer peptides (ACPs) [16]. These peptides exhibit unique advantages over traditional chemotherapy agents, including limited toxicity toward normal mammalian cells, low probability of resistance generation, and the ability to eliminate cancer cells through different mechanisms without generating significant adverse effects [17,18]. HDPs can interact specifically with the negatively charged membranes of microorganisms and cancer cells, resulting in alterations in their permeability and integrity that may ultimately lead to cellular lysis or necrosis, which constitute the primary activity of these peptides as antimicrobials and anti-cancer agents [19,20]. The outer layer of the plasma membrane of cancer cells contains high proportions of the negatively charged lipid phosphatidylserine (PS), in contrast to normal cells that only present it in the inner layer of the membrane [21]. Some cancer cells aberrantly externalize phosphatidylethanolamine (PE), which increases membrane fluidity and makes cancer cells’ membranes more susceptible to the membrane-disruptive action of HDPs [20,21]. The high expression of O-glycosylated mucins and sialylated gangliosides also increases the negative charge of the plasma membrane of cancer cells, promoting the interaction of cationic HDPs [21,22]. Furthermore, HDPs can induce other activities in cancer cells, including the induction of apoptosis, inhibition of angiogenesis, inhibition of essential proteins, and modulation of immune response [14,15,23,24].

**Figure 1:**
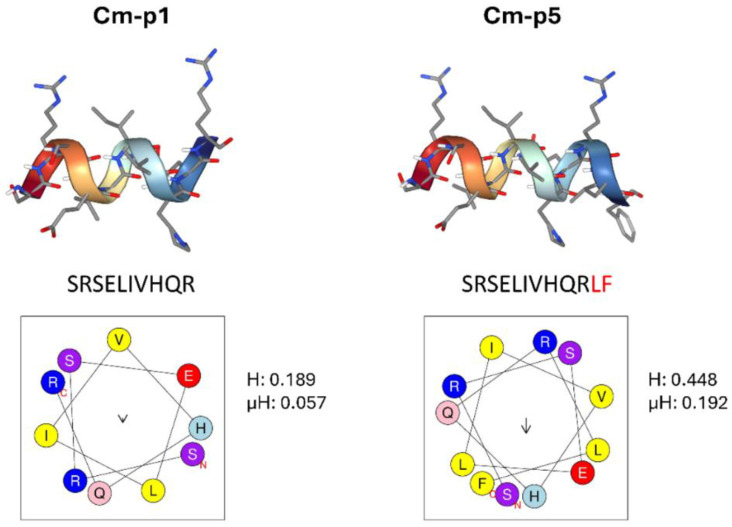
3D structure of Cm-p1 (left panel) and Cm-p5 (right panel) predicted by PEP-FOLD4 Server. Both peptides adopted α-helical conformations; blue represents nitrogen atoms, and red represents oxygen atoms. The two extra aminoacids in the sequence of Cm-p5 are highlighted in red. Below each sequence is the corresponding helix projection obtained with the HeliQuest server. Positively charged residues are represented in blue and hydrophobic residues in yellow.

HDPs represent a group of evolutionarily conserved molecules of innate immunity that can be found in a wide range of organisms, especially in invertebrates, which lack adaptive immune responses and produce a large number of HDPs as a primary defense mechanism [25,26]. Marine invertebrates are considered an excellent source of biologically active peptides due to the constant biological challenge of the oceanic environment, altered mainly by human action [26,27]. Furthermore, the evolution of these organisms in saline conditions rendered peptides resistant to high salt concentrations, one of the most common causes of peptide activity inhibition [28,29]. In this sense, our research group isolated several peptides from marine invertebrates with remarkable antimicrobial activity, and these have been modified to obtain more effective variants. Cm-p5 (SRSELIVHQRLF-NH_2_) is a hydrophobic peptide with a net positive charge of +2 at physiological pH derived from the natural peptide Cm-p1 (Figure 1), originally isolated from the coastal snail, *Cenchritis muricatus* (*Gastropoda: Littorinidae*), which has shown antifungal activity against *Candida albicans*, *Candida auris,* and *Candida parapsilosis*, including resistant strains [30]. Cm-p5 has been characterized by circular dichroism and NMR spectroscopy, revealing an amphipathic α-helical structure under conditions that mimic biological membranes, as well as a tendency towards a random structure in aqueous solutions [31]. Lopez-Abarrategui demonstrated, by isothermal titration calorimetry, the ability of this peptide to interact with high affinity with PS, PE, and ergosterol [30]. Interestingly, the interaction with PS was the strongest of all, suggesting the possibility that Cm-p5 could interact with tumor membranes and exhibit mechanisms similar to those observed in *Candida* [30]. Although progress has been made in the structural and functional characterization of Cm-p5 as an antimicrobial, the potential of this peptide as a possible anti-cancer drug remains unexplored. Therefore, this study aims to evaluate the anti-cancer activity of the antimicrobial peptide Cm-p5 and to propose a preliminary mechanism of action of this peptide in malignant cells based on the applied concentration.

## 2. Results

### 2.1. Cm-p5 Didn’t Affect the Integrity of Human Erythrocytes 

**Figure 2:**
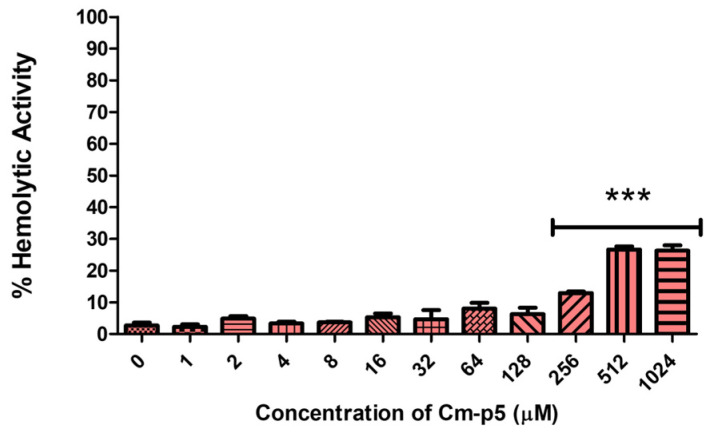
The percentage of hemolytic activity of Cm-p5 in hRBC determined by hemoglobin release. A range of peptide concentrations was evaluated against hRBC for 2 h. PBS and Triton X-100 at 0.1% were used as negative and positive controls, respectively. The results are representative of three independent experiments, analyzed by one-way ANOVA with α = 0.05 to assess the differences between the peptide concentrations and the negative control (*** *p* < 0.001).

One of the most common therapeutic problems of ACPs is their hemolytic activity, especially for highly hydrophobic peptides with a membranolytic effect [14]. Although the template peptide Cm-p1 doesn’t exhibit hemolytic activity towards human red blood cells (hRBC), the risk is higher for Cm-p5 due to the addition of two amino acids that increased its hydrophobicity [32]. Even so, the hemolysis assay revealed that Cm-p5 exhibits no significant hemolytic activi-ty against hRBC at a wide range of concentrations (Figure 2). Only at the high concentrations of 1024 μM, 512 μM, and 256 μM, a moderate hemolytic activity was detected. However, this activity was under 30%, supporting the non-hemolytic profile of Cm-p5.

### 2.2. Cm-p5 Reduces the Viability of A375 Melanoma Cells Without Affecting Non-Malignant WI-38 Fibroblasts

**Figure 3:**
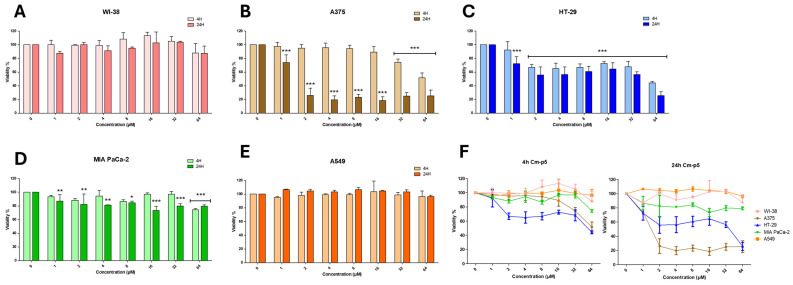
Effects of Cm-p5 on cell viability of fetal lung fibroblasts WI-38 (**A**), malignant melanoma A375 (**B**), colon carcinoma HT-29 (**C**), pancreatic carcinoma MIA PaCa-2 (**D**), and epithelial cell lung carcinoma A549 (**E**), determined by MTT assay at 4 and 24 h of incubation. (**F**) Comparison of the effect of Cm-p5 in each cell line at both incubation times. Cells treated with culture medium, represented by 0 µM, were used as a negative control and taken as 100% viability. The results shown are representative of three independent experiments and were analyzed using one-way ANOVA with α = 0.05 to assess differences between peptide concentrations and the negative control for each cell line (* *p* < 0.05, ** *p* < 0.01, and *** *p* < 0.001).

The cytotoxic activity of Cm-p5 was evaluated using an MTT assay in non-malignant and cancer cell lines after treatment of the cells with varying peptide concentrations for 4 and 24 h. As can be observed in Figure 3A, the peptide Cm-p5 does not produce a significant decrease in the viability of WI-38 cells (human fetal lung epithelial fibroblasts) at all concentrations tested. The WI-38 cell line is widely used to evaluate the toxicity of potentially harmful molecules due to its sensitivity, fetal tissue origin, and diploid karyology [33]. Therefore, the absence of toxicity in this cell line constitutes a safety criterion for using Cm-p5 as a potential anti-cancer drug. 

In the case of the A375 melanoma cell line, cytotoxic activity was only observed at 4 h with 32 and 64 µM of Cm-p5, unlike at 24 h of incubation, where viability decreased dramatically in the range of 2 to 64 µM without significant differences between concentrations (Figure 3B). This behavior is considerably atypical of the effects of classical membranolytic or pore-forming peptides that rapidly eliminate cancer cells even at low concentrations, suggesting that Cm-p5 follows a different mechanism of action. The change in IC50 (half-maximal inhibitory concentration) of Cm-p5 in A375 cells from 68.89 µM at 4 h to 1.58 µM at 24 h (Table S1) suggests that cytotoxicity at low concentrations may be the result of cumulative damage over time. However, the activity at 4 h with 32 and 64 µM also indicates different effects on A375 cells between high and low concentrations of Cm-p5, which can be related to the amount of peptide required to initiate cell damage. In contrast, Cm-p5 decreases the viability of HT-29 cells by approximately 60% in the range of 2 to 32 µM at 4 h, with a sharp drop at 64 µM in a dose-dependent manner (Figure 3C). This effect is slightly increased after 24 h, without substantial changes in the range of 2 to 32 µM, suggesting that the peptide only acts in the first hours of treatment, possibly through membrane disruption. In the pancreatic carcinoma cell line MIA PaCa-2, Cm-p5 exhibited cytotoxicity only at high concentrations (32–64 µM), particularly at 24 h (Figure 3D). Nevertheless, the activity was considerably weaker than in HT-29 and A375 cells. Additionally, no significant decrease in viability was observed in A549 cells across the en-tire concentration range at both incubation times (Figure 3E). The comparison of the cytotoxic activity of Cm-p5 at 4 h and 24 h in the mentioned cell lines (Figure 3F) highlights the specificity of Cm-p5 for melanoma and colon carcinoma cells. Among the evaluated cell lines, A375 exhibited the highest sensitivity to Cm-p5, with an IC50 value of 1.58 ± 1.25 µM. Consequently, it was selected for further investigation into the underlying anti-cancer mechanisms of Cm-p5.

### 2.3. Cm-p5 Causes Cell Aggregation, Swelling, and Chromatin Condensation in A375 Cells, Without Nuclear Fragmentation 

**Figure 4:**
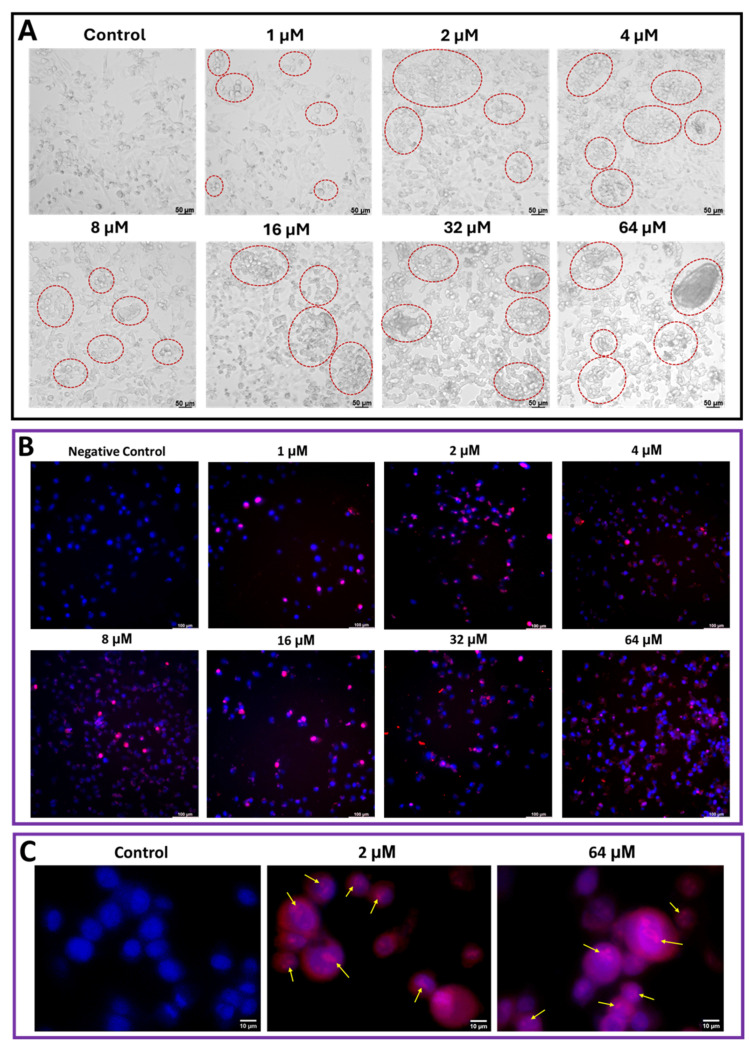
Effects of Cm-p5 on the morphology of A375 melanoma cells. (**A**) Photomicrographs of cells treated with (1–64 µM) of Cm-p5 for 24 h at 200 magnification. The observed alterations include changes in cell size, aggregation, syncytia formation, and loss of adhesion capacity, highlighted by the red circles. (**B**) Merge of fluorescence micrographs of DAPI and PI-labeled cells exposed to different concentrations of Cm-p5 for 24 h at 200× magnification, with altered nuclear morphology and disrupted membranes. In all cases, cells treated with DMEM medium were used as negative controls. (**C**) Merge of fluorescence micrographs of DAPI/PI-labeled cells treated with 2 µM and 64 µM (as representatives of low and high concentrations, respectively) of Cm-p5 for 24 h at 630× magnification. The yellow arrows indicate zones with condensed chromatin.

The morphology of A375 melanoma cells was analyzed by bright field microscopy after 24 h of treatment to confirm possible alterations due to the action of the peptide (Figure 4A). In general, the peptide causes cell swelling and the formation of large aggregates or syncytia. In addition, as the concentration of Cm-p5 increased, a greater number of rounded and detached cells were observed, which reflects an impairment in their adhesion capacity. Therefore, the effect on A375 showed a duality in relation to the concentration of Cm-p5, with the formation of aggregates in the range of 1-16 µM and a severe swelling at higher concentrations such as 32 and 64 µM, which can be associated with a membranolytic or necrotic mechanism, although at low concentrations it also causes a slight swelling of the cells.

DAPI is a fluorescent probe used to visualize nuclear morphology, while PI is a fluorophore used to observe membrane disruption and chromatin condensation. Both fluorescent probes bind to DNA, and their joint application generates a contrast between live and dead cells since PI can only cross the membrane when it is affected. Live cells appear marked only by DAPI, and dead or damaged cells are marked with both fluorophores, showing a characteristic purple or pink color due to the fusion of both labels. This experiment was performed in A375 cells after 24 h of treatment with Cm-p5 (Figure 4B). As expected, double labeling was observed in A375 cells at all peptide concentrations, demonstrating the ability of Cm-p5 to disrupt cytoplasmic membrane integrity even at low concentrations. Interestingly, some isolated and large syncytia, as well as intense PI labeling, were observed in the cells treated with 32 and 64 µM of Cm-p5 due to severe damage to the cytoplasmic membrane. Figure 4C shows a swollen nucleus without nuclear fragmentation, and the presence of intranuclear granules representative of areas with highly condensed chromatin in cells treated with 2 and 64 µM of Cm-p5, which are commonly associated with dead cells or cells in a late stage of apoptosis [34,35,36]. 

### 2.4. Cm-p5 Induces Rapid Depolarization of Cytoplasmic Membranes of A375 Cells 

The effects of the peptide Cm-p5 on the membrane potential of A375 cells were determined using the voltage-sensitive dye 3,3′-Dipropylthiadicarbocyanine iodide [DiSC3 (5)]. DiSC3 (5) is a cationic, membrane-permeable fluorescent dye that can accumulate in polarized cells and acts as a potentiometric probe [37,38].

**Figure 5:**
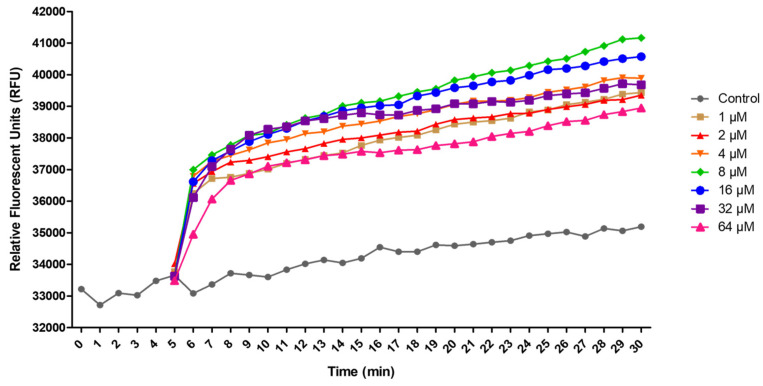
Membrane depolarization of A375 cells caused by Cm-p5 for 25 min was determined with DiSC3 (5). The graph illustrates the changes in fluorescence intensity of DiSC3 (5) at varying concentrations of Cm-p5. Cells treated with PBS were used as negative controls. The results represent the mean of three independent experiments performed in triplicate; error bars were omitted for clarity.

Cm-p5 induced a fast depolarization of A375 cells that increased over time for each concentration, which can be a direct consequence of the interaction of the peptide with cytoplasmic membranes and their subsequent permeabilization (Figure 5). The depolarizing effect increased from 1 µM to a maximum reached at 8 µM. Nevertheless, the effect is reduced at higher peptide concentrations, with the lowest fluorescent signal observed at 64 µM, but still higher than that of the negative control. This result suggests that at low or moder-ate concentrations, the peptide Cm-p5 can induce a controlled depolarization. In contrast, at higher concentrations (32–64 µM), it can affect the integrity of the cellular membrane or even cause toxicity, leading to a partial loss of the membrane potential. Furthermore, the initial permeabilization and disruption of the cytoplasmic membrane by the peptide may cause leakage of the fluorescent probe or impede its proper accumulation and distribution within the cell. 

### 2.5. The Peptide Cm-P5 Is Localized in the Membrane of A375 Cells and in Debris Clusters

**Figure 6:**
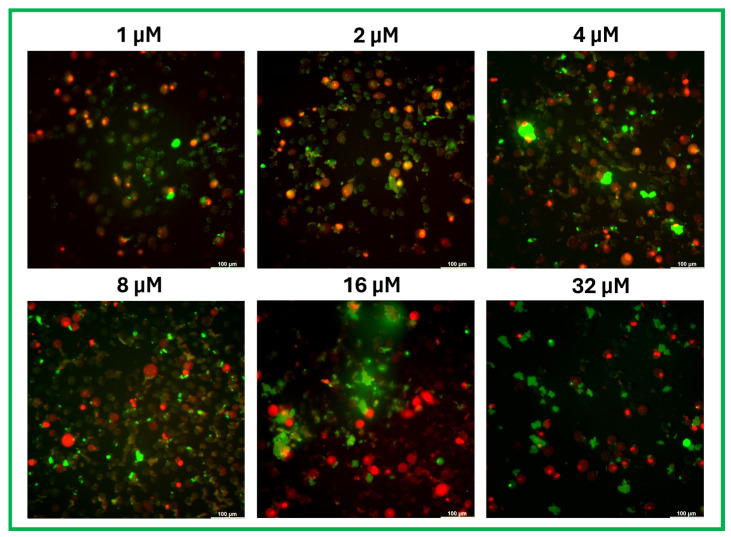
Staining with Cm-p5-FITC and PI in A375 cells. Merged fluorescence micrographs of cells exposed to different concentrations of FITC-labeled peptide for 24 h at 200× magnification. PI staining was also performed to check the membrane integrity of the cells interacting with Cm-p5.

The interaction of Cm-p5 with melanoma cells and its cellular localization were confirmed by fluorescence microscopy using a FITC-conjugated peptide. Figure 6 shows the peptide localization in A375 cells at several concentrations after 24 h of incubation. Additionally, PI staining facilitates the identification of damaged cells. It differentiates them from the nuclear labeling shown by PI, as opposed to the labeling provided by the FITC-conjugated peptide. At concentrations of 32 and 16 µM, the peptide was concentrated in debris clusters and the membranes of severely damaged cells, confirming the hypothesis of a membranolytic effect at high concentrations and the capacity of Cm-p5 to remain attached to the cells even after the cytotoxic action. Below 8 µM, the peptide was homogenously distributed through the membrane of A375 cells, and most PI-positive cells were also labeled with the conjugate, which reflects the relationship between the interaction of Cm-p5 with the membrane of melanoma cells and its disruption. It is notable that the presence of cells labeled with Cm-p5-FITC and slight or no labeling with PI at 2 and 1 µM, possibly corresponding to those cells that, due to the low concentration of peptide, are not yet entirely compromised. In contrast, many cells with the highest PI labeling exhibited large nuclei and low conjugate incorporation, which could be associated with an indirect action that does not require interaction with the cytoplasmic membrane.

### 2.6. Cm-p5 Induces Cell Death in Melanoma Cells 

**Figure 7:**
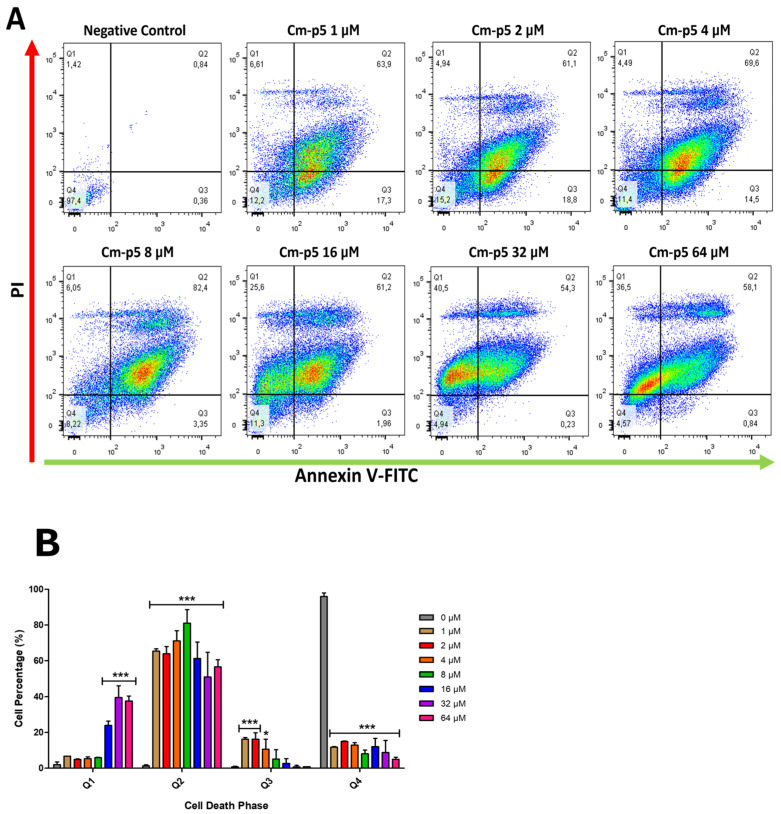
Determination of the capacity of Cm-p5 to induce cell death in A375 cells by flow cytometry with Annexin V-FITC/PI. (**A**) Density graphs of the double labeling Annexin V-FITC/PI in cells treated with 1 to 64 µM of Cm-p5 for 24 h. In all plots, the Y axis represents the labeling with PI, and the X axis represents the labeling with Annexin V-FITC. Cells treated with PBS were used as negative controls. The cells are grouped into 4 quadrants: Q1 (dead cells), Q2 (late stages of apoptosis or necrotic), Q3 (early stages of apoptosis), and Q4 (normal and healthy cells). (**B**) Statistical analysis of the percentage of cells in each quadrant for all the concentrations of Cm-p5. The histograms represent the average ± SD of three independent experiments (* *p* < 0.05, *** *p* < 0.001).

PS exposure to the outer face of the cytoplasmic membrane is a typical indicator of necrosis or early stages of apoptosis [39]. In Figure 7, the percentages of A375 cells labeled with Annexin V-FITC and PI are represented in graphs for 24 h of treatment with Cm-p5 at concentrations ranging from 1 to 64 µM. The quadrants were established based on the signals obtained from the negative controls stained with Annexin V-FITC and PI, such that most of their events were considered live cells. The signals received in these controls represent both the intrinsic fluorescence of the cells and the basal exposure of PS in tumor cells and cells damaged during processing, which guarantees that the labeling observed in the other quadrants is exclusively associated with the effects of the treatments. Quadrants Q1, Q2, Q3, and Q4 correspond to dead cells, necrotic or late stages of apoptosis, cells exhibiting PS or in early stages of apoptosis, and normal cells, respectively. 

Only a low percentage of cells in the early phase of apoptosis was observed at con-centrations below 4 µM. A significant number of dead cells was not detected at this concentration. On the other hand, a high percentage of cells were in a necrotic state or a late phase of apoptosis, evidenced by the double labeling. This tendency towards the late phase was maintained at 8 µM, reinforced by the loss of cells in the early phase at the previous concentration, with rela-tively few dead cells. The inflection point was found at 16 µM, where the number of dead cells begins to increase, although it remains lower than that of necrotic cells or in the late phase of apoptosis. At 32 and 64 µM, the transition to necrosis is even greater, with a percentage of dead cells similar to that of late-phase cells. Curiously, a group of events isolated from the main cluster was observed at every concentration, primarily manifesting as necrotic cells or in the late phase of apoptosis, characterized by high PI labeling. 

### 2.7. Cm-p5 Alters the Cell Cycle of A375 Cells and Promotes DNA Fragmentation

**Figure 8:**
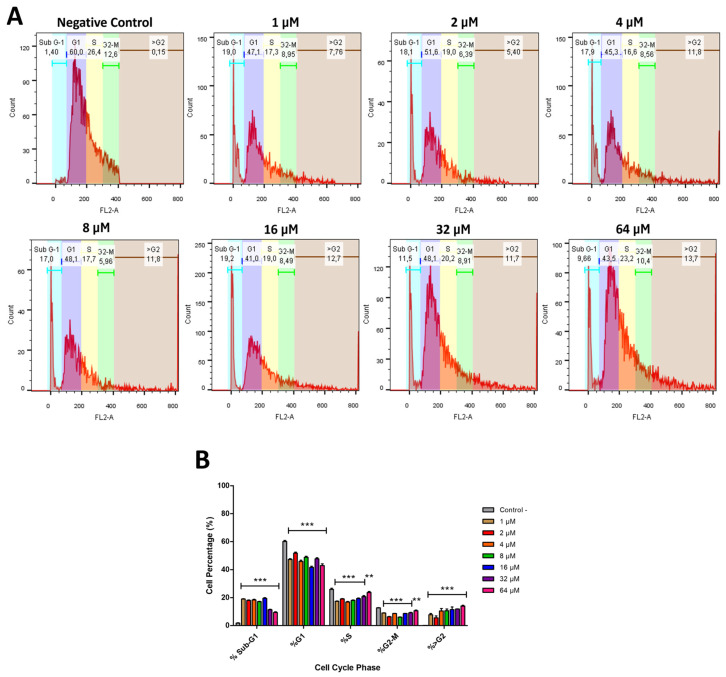
Cell cycle analysis of A375 cells treated with Cm-p5 for 24 h. Based on the signal given by PI labeling, cell cycle phases and the percentages of cells in each phase were identified. In (**A**), five main phases shown highlighted with colors: Sub-G1 (turquoise), G1 (blue), S (yellow), G2-M (green), and >G2 (brown). A375 cells treated with DMEM medium were used as negative control. In all graphs, the Y axis represents the cell count, and the X axis represents the PI labeling on a linear scale. (**B**) Mean percentage of cells ± SD in each cell cycle phase for all conditions. The graphs represent results obtained from three independent experiments, and the differences between treatments and negative controls were analyzed by one-way ANOVAs (** *p* < 0.01, and *** *p* < 0.001).

Analysis of the cell cycle after treatment with the peptide could help explain whether the effects observed in previous experiments result from a possible arrest of the cell cycle due to apoptosis induction. This was assessed by staining the DNA content with PI in ethanol-fixed cells, with subsequent analysis by flow cytometry. This method identifies apoptotic cells as a Sub-G1 fractional population with low DNA content due to the DNA fragmentation that typically occurs during apoptosis [40]. Figure 8A shows the graphs corresponding to the percentages of A375 cells treated with increasing concentrations of Cm-p5 in each cell cycle phase. The results do not indicate a complete arrest of the cell cycle, but rather a significant de-crease in the number of cells in the G1, S, and G2 phases, along with the presence of cells in the Sub-G1 population, as shown in Figure 8B. The percentage of Sub-G1 cells remains similar at concentrations ranging from 1 to 16 µM, while it decreases at 32 and 64 µM, possibly due to an increase in the number of dead cells at higher concentrations. Additionally, the appearance of >G2 populations was verified at all concentrations, which correspond to multinucleated cells, resulting from the fusion of several cells and, therefore, with a high DNA content and PI labeling [41,42]. These syncytia were observed in microscopy assays and Annexin-V FITC/PI stainings as a consequence of the membrane-permeabilizing effect of Cm-p5. 

### 2.8. The Proliferation Capacity of A375 Cells Is Affected After the Treatment with Cm-p5

Once the alterations caused in A375 cells by Cm-p5 were confirmed, the capacity of these tumor cells to recover from the damage was evaluated over 7 days with Carboxyfluoreszin-Succinimidyl-Ester (CFSE) staining. During this period, the cells were kept in a fresh culture medium fully supplemented to promote their proliferation in an adequate environment that simulates the conditions of tumor recovery. The recovery capacity was determined using the degree of cell division as an indicator, defined by the intensity of the labeling due to the presence of CFSE. This fluorophore can internalize and bind strongly to the DNA of the cells. With each division, the fluorescence intensity is equally halved, giving rise to several intensity peaks in the flow cytometry [43]. The peaks with the highest fluorescence intensity correspond to cells that did not divide at all, and the peaks with the lowest intensity correspond to cells with different degrees of division. 

**Figure 9:**
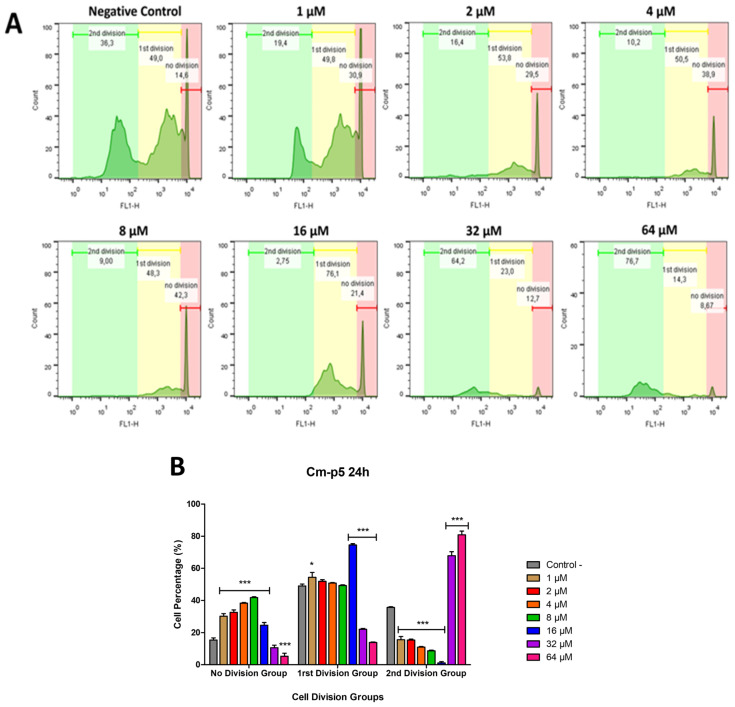
Evaluation of the proliferative capacity of A375 cells 7 days after the treatment with different concentrations of Cm-p5 for 24 h by CFSE assay. Three groups were identified based on the fluorescence intensity, indicating the degree of cell division. A higher fluorescence value indicates a lower proliferation and vice versa. The groups are represented with colors from lowest to highest according to the division order: no division (red), 1st division group (yellow), 2nd division group (green). (**A**) Histograms correspond to the negative control and each peptide concentration, where the Y axis represents the cell count, and the X axis represents the CFSE labeling. Cells treated with culture medium were used as negative controls. (**B**) Mean ± SD percentage of cells in each division group for all conditions, obtained from three independent experiments. Differences between treatments with different concentrations of Cm-p5 and the negative control in each group were analyzed using one-way ANOVAs (* *p* < 0.05 and *** *p* < 0.001).

In A375 cells, three main populations were identified from lowest to highest according to the degree of division: non-division (red), 1st division group (yellow), and 2nd division group (green) (Figure 9A). Cells treated with culture medium (Negative Control) were taken as a reference for proliferation, where most of the events are observed in the first and second division groups. At all Cm-p5 concentrations, the decrease in the number of detected cells is remarkable.

At low concentrations (1–8 µM), approximately 10 to 20% of cells were detected in the second division group, with the highest percentage at the lowest peptide concentration. Most of the remaining cells stayed between the non-division and first division groups. In contrast, at 16 µM, the percentage of cells in the second division group was minimal, and most of the cells were found in the first division group, with fewer cells in the non-division group. On the other hand, at 32 and 64 µM, few cells were found in both the non-division and first division groups, with a marked increase in cells in the second division group. 

These results demonstrate that the best antiproliferative effects are obtained with low concentrations of Cm-p5 (1–8 µM), which decrease at higher concentrations such as 32 and 64 µM, with 16 µM as an intermediate point in this transition (Figure 9B). Although the treatment with 32 and 64 µM at first glance might seem counterproductive, the cell counts obtained were excessively low because of the potent activity of Cm-p5 at these concentrations. This dual behavior in terms of affecting cell proliferation may be associated with the differences in the mechanisms of action observed for each concentration.

### 2.9. The Treatment with Cm-p5 Stimulates Intracellular ROS Production in A375 Cells

ROS are metabolites of oxidative respiration, mainly generated within the mitochondria, that are reduced by specific enzymes to avoid harmful effects [44]. Whether low ROS levels can activate signals in response to stress or damage to promote proliferation and cell survival, ROS accumulation leads to DNA damage, protein and lipid oxidation, and even cell death [45]. Intracellular ROS production was evaluated in A375 cells treated with Cm-p5 to determine if the membrane damage caused by this peptide can trigger this cellular response and elucidate its possible role in the induction of cell death. 

**Figure 10:**
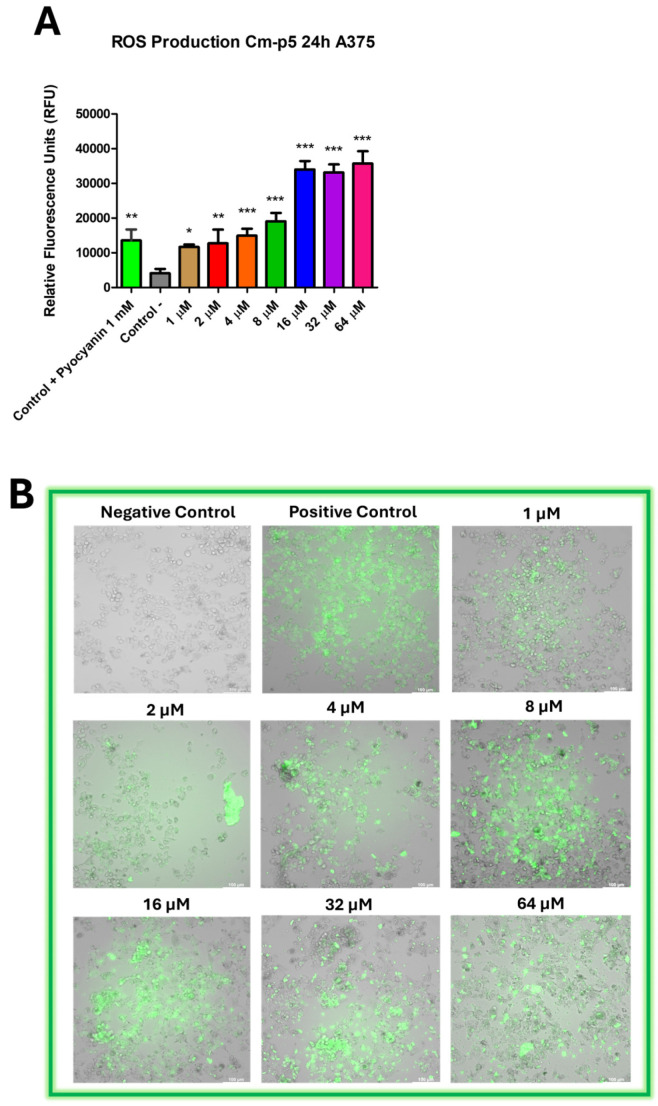
Evaluation of intracellular ROS production in A375 cells in response to Cm-p5 treatment for 24 h. (**A**) ROS levels were measured using the H2DCFDA-Cellular ROS assay. Cells treated with medium and the ROS inducer pyocyanin at 1 mM for 2 h were used as negative and positive controls, respectively. The graph shows the mean ± SD of Relative Fluorescence Units obtained from three independent experiments and analyzed by one-way ANOVA (* *p* < 0.05, ** *p* < 0.01, and *** *p* < 0.001). (**B**) Merged fluorescence micrographs of A375 cells treated with Cm-p5 show intracellular ROS in correspondence with morphology impairment.

Cm-p5 induced the production of ROS in A375 cells at 24 h of incubation (Figure 10A), especially at high concentrations (16–64 µM), at which the peptide exhibits stronger membranolytic activity. This reveals a direct relation between the severity of the membrane damage generated by Cm-p5 and the amounts of ROS produced by the cells. Moreover, significant signals were obtained at the range of 1 to 8 µM, demonstrating that the sustained permeabilizing effect of Cm-p5 observed at these low concentrations is enough to induce ROS production. Fluorescence microscopy images support these results (Figure 10B). Most cells showed the presence of intracellular ROS, independently of the peptide concentration applied. Still, it’s noteworthy that the brighter fluorescent labeling was detected in swollen cells and cell clusters generated by the permeabilization of cytoplasmic membranes.

### 2.10. Caspase-3/7 Remains Active in A375 Cells After 24 h Treatment with Cm-p5 

**Figure 11:**
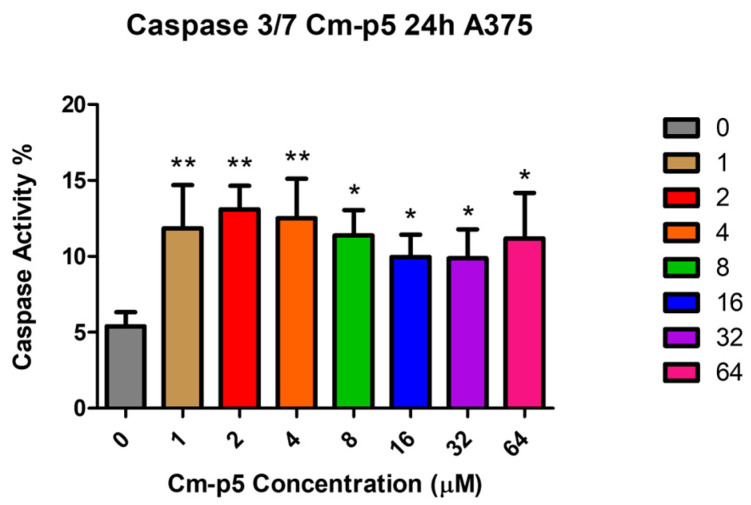
Percentage of caspase-3/7 activity in A375 cells treated with several concentrations of Cm-p5 for 24 h. The graph represents results obtained from three independent experiments, and the differences between treatments and negative controls were analyzed by one-way ANOVAs (* *p* < 0.05, ** *p* < 0.01).

The capacity of Cm-p5 to induce apoptosis in A375 cells was determined by the evaluation of effector caspase-3/7 activity, a distinctive marker of this process, using a commercial colorimetric kit (Figure 11). Caspase activity did not surpass 15 % at any concentration but was slightly higher at low concentrations of Cm-p5 (1–4 µM), which aligns with the results obtained in the cell cycle analyses. However, caspase activity was measured at 24 h, when most of the A375 cells are in the late stage of apoptosis or have already died, according to the results from the Annexin-V FITC/PI assay. 

## 3. Discussion

Most cancer deaths are associated with complications from the development of metastases in critical organs [4,46]. Unfortunately, in many severe cases, chemotherapy regimens involve the frequent and prolonged use of several cytostatic drugs, which also contribute to the gradual deterioration of the patient’s health [5,47]. This situation worsens when considering the intersection between cancer and infections, evidenced by the number of new cases and deaths related to the treatment of chronic fungal or bacterial infections [4,48,49]. Patients suffering from chronic infections are more susceptible to neoplastic diseases due to the weakening of the immune system, unable to respond efficiently against pathogens, and the emergence of cancer cells simultaneously [50,51]. Likewise, immunosuppression can be the result of aggressive chemotherapy or radiotherapy, which increases the susceptibility of patients to infections [12,52,53]. In addition, continuous exposure to infectious agents leads to inflammation, thus contributing to cancer development [54,55]. Although there is a wide variety of drugs available, cancer and infection treatment have a common problem: the emergence of resistance to multiple drugs [5,6,11,50]. This poses a complex scenario, where it is imperative to search for new antitumor and antimicrobial drugs with greater selectivity and different mechanisms of action that do not depend on the interaction with a specific molecule in the target cells, like those currently used in therapy [6,18,23].

The antimicrobial peptide Cm-p5 was obtained as part of a rational design, adding two aminoacids to the sequence of the natural peptide Cm-p1 [30,32]. This modification enhanced the antifungal activity of Cm-p5 and its capacity to interact with negatively charged phospholipids [30]. Molecular dynamic simulations performed with PS bilayers indicate that Cm-p5 follows a carpet model since no penetration of the peptide was observed, and it remained parallel to the membranes, interacting with the polar heads of the lipids [30]. This mode of interaction was confirmed in *Candida albicans*, revealing a membrane perturbation mechanism of action without pore formation [30,56]. A similar behavior could be expected in cancer cell membranes with a significant content of PS [21,57]. It is also necessary to consider the possible interactions of Cm-p5 with other anionic molecules such as glycoproteins, mucins, gangliosides, and other lipids like PE and cholesterol. 

The peptide Cm-p5 exhibits a dual mechanism of action, dependent on both concen-tration and incubation time, in A375 cells. At low concentrations, the effects include mem-brane depolarization, sustained permeabilization, induction of apoptosis, ROS accumula-tion, and inhibition of cell proliferation since the amounts of peptide are insufficient to induce a membranolytic mechanism. In contrast, at high concentrations, the amount of peptide would sufficient to form complexes in the cell membrane and cause cell death through rapid mechanisms, such as membrane disruption or necrosis, resulting in a high production of ROS. A375 melanoma cells have a high content of PS and cholesterol in their plasma membrane, which may explain the interaction of Cm-p5 with these cells and the temporal delay in its action, respectively [58]. The inhibitory effect of cholesterol on the activity of HDPs was previously described by Matsuzaki et al. as a way in which host cells protect themselves from the membranolytic action of their peptides, and has been demonstrated with several HDPs, such as LL-37, gramicidin S, and dermaseptin DD K [59,60,61,62]. For a peptide that interacts with a carpet model like Cm-p5, reaching a threshold concentration over the membrane is necessary to generate micelles through a detergent-type action, especially in rigid membranes with high cholesterol contents [63,64,65,66,67]. Nevertheless, if the peptide concentration is below this threshold, more interaction time will be required to generate significant membrane damage that can accumulate and lead to cell death [63,68,69]. Therefore, at low concentrations of Cm-p5, there is a slight loss in immediate activity but a gain in cytostatic capacity and cell death induction in the long term, which represents an advantage for the design of possible treatment schemes, given the high cost of synthesis, extension of the time between doses and lower exposure of the patient to the peptide [14,70,71]. The antiproliferative activity of Cm-p5 is exclusive to tumor cells, as this effect is not observed in *Candida albicans* cells, which begin to recover and proliferate after 24 h of continuous treatment with the MIC of the peptide [72]. The potent cytotoxic activity of Cm-p5 against A375 cells places it between the most active ACPs reported to date that are derived from marine invertebrates [26,69,73,74]. Among them, Kahalalide F isolated from the mollusk *Elysia rufescen* is distinguished by its necrotic activity against several cancer cell lines with IC50 ranging from 0.2 to 10 µM [75,76,77]. Similarly, the alpha helical peptide P6 isolated from the bivalve *Arca inflata* induces apoptosis and ROS production in human colorectal carcinoma cell lines, with IC50 ranging from 4 to 11 µM [78]. Several ACPs from non-marine organisms are well characterized and serve as benchmarks for activity and mechanism of action [14,16,18,19,24]. The peptide gomesin, isolated from the hemocytes of the spider *Acanthoscurria gomesiana*, exhibits cytotoxic activity following a carpet model, with an IC50 value below 5 µM in several melanoma cell lines [79,80]. The alpha helical peptide mellitin induces apoptosis and inhibits the growth of melanoma cells, with IC50 ranging from 3 to 4.5 µM [81]. Compared to these peptides, Cm-p5 stands out by its short sequence, linear structure, and selectivity.

Cellular stress caused by the progressive action of Cm-p5 on the membrane could be the primary trigger of cell death in A375, via mitochondrial membrane permeabilization, metabolic alterations, cytoskeleton disturbance, and ROS overproduction [82,83,84]. The accumulation of ROS can induce further damage to the cells, creating a feedback loop that results in more ROS and the promotion of several forms of cell death, including apoptosis and necroptosis [45,84]. It is noteworthy that the presented results are not conclusive enough to rule out the induction of apoptosis by Cm-p5, as the experiments were performed after 24 h of treatment, when most melanoma cells entered the late stage of apoptosis or died. At this point, the detected caspase activity may be just a remnant of an early process. Even though chromatin condensation is typically associated with apoptosis, it also arises as a consequence of the loss of structural integrity in the nuclear lamina and nuclear matrix [24,35,36]. Nevertheless, if apoptosis was the main form of cell death induced by the action of Cm-p5, a higher percentage of cells in the Sub-G1 phase with degraded DNA would be expected, at least comparable to the percentages of cells in the late stage. To confirm the induction of apoptosis, caspase activity must be monitored over time (2 h, 4 h, 8 h, 12 h, and 24 h) together with the appearance of early and intermediate apoptosis markers, such as PS externalization, mitochondrial membrane potential loss, cytochrome C release, and PARP cleavage [39,40,85,86]. Additionally, A375 cells can be treated with Cm-p5 in the presence and absence of the pan-caspase inhibitor zVAD-FMK to evaluate if cell death is prevented, thus demonstrating the participation of apoptosis in the peptide’s mechanism of action [39,40]. Although A375 cells treated with Cm-p5 exhibited typical markers of necrosis, the possible role of necroptosis should not be underestimated due to their similarities and potential for the treatment of apoptosis-resistant tumors [45,87]. Necroptosis is a caspase-independent form of regulated necrosis induced by the recognition of tumor necrosis factor cytokines, metabolic stress, and virus infection [88,89]. The necroptotic pathway involves the formation of a complex between receptor-interacting protein kinase 1 (RIPK1), RIPK3, and mixed lineage kinase domain-like protein (MLKL), which promotes ROS production, lipid and protein oxidation, membrane permeabilization, and cytosolic ATP reduction [45,88,90,91,92]. Although RIPK1 and MLKL levels are homogeneous in most melanoma cell lines, the expression of RIPK3 is extremely low, which has been associated with the absence of necroptosis [93,94,95]. Even so, there are certain reports of necroptosis induction in A375 cells by drugs such as sanguilutine, evodiamine, and the naphthyridine derivative 3u [96,97,98]. The necroptotic activity of Cm-p5 could be confirmed by evaluating RPK1, RIPK3, and MLKL expression in A375 cells or by using necroptosis inhibitors, such as necrostatin-1 and necrosulfonamide [94,97,99]. In contrast to apoptosis, which is considered an immunologically silent process, necroptosis and necrosis promote pro-inflammatory signaling and antitumor immunity by releasing danger-associated molecular patterns (DAMPs) [91,100,101,102]. Further studies are required to determine the capacity of Cm-p5 to induce antitumor immunological responses in vivo, similar to other α-helical ACPs with necrotic activity [64,103,104,105,106,107,108,109,110,111,112,113,114,115,116,117].

The remarkable activity of the Cm-p5 peptide in A375 cells highlights its potential for treating malignant melanoma, the most severe form of skin cancer, which accounts for 80% of skin cancer-related deaths [118,119]. One of the major problems is the low availability of therapeutic drugs [120]. To date, the FDA-approved agents include cytostatics like DTIC, immunotherapeutics such as IL-2, ipilimumab (an anti-CTLA4 antibody), nivolumab and pembrolizumab (PD-1 blocking antibodies), the BRAF inhibitors vemurafenib and dabrafenib, and the MEK1/2 inhibitors binimetinib and trametinib [121,122,123,124,125,126,127,128,129,130]. All these drugs face problems such as the development of resistance, adverse effects, mutation dependence, and poor responses against advanced tumors, which underscores the severe need for new drugs for the treatment of malignant melanomas [131,132,133,134,135]. On the other hand, most HDPs approved for clinical use are applied topically due to their poor oral bioavailability, which results from their susceptibility to serum proteases and poor intestinal membrane penetration [136,137]. In this sense, malignant melanoma is an attractive target for the application of ACPs, as many of the complications and disadvantages associated with administering these mole-cules can be avoided, substantially increasing the chances of successful treatment [70,71]. 

## 4. Materials and Methods

### 4.1. Prediction of Peptide Structure 

The helical wheel projection, hydrophobicity, and hydrophobicity moment of Cm-p5 and Cm-p1 were predicted using the helical wheel predictor HeliQuest (http://heliquest.ipmc.cnrs.fr/cgibn/ComputParam.py) (accessed on 9 June 2025). The three-dimensional structure model of the peptides Cm-p1 and Cm-p5 was generated using the online Peptide Structure Prediction Server PEP-FOLD4 (https://bioserv.rpbs.univ-paris-diderot.fr/services/PEP-FOLD4/#overview) (accessed on 9 June 2025).

### 4.2. Peptide Synthesis

The peptide Cm-p5 was synthesized in solid phase using the Fmoc/tBu methodology and the Rink linker for the generation of C-terminal amidated peptide and purified by reversed-phase high-performance liquid chromatography (RP-HPLC) using an acetonitrile/H2O-TFA gradient to over 95% purity. To generate a fluorescently labeled peptide, 5(6)-carboxyfluorescein was coupled to the N-terminus of Cm-p5 after the coupling of the complete sequence employing a DIC/Oxyma as coupling mixture in DMF. The molecular mass was confirmed by electrospray ionization mass spectrometry (ESI-MS) (Figures S1 and S2) (Peptide synthetic Group, Biomedical Research Division, CIGB, Havana, Cuba) [30].

### 4.3. Human Cells and Culture Conditions

The human cancer cell lines, A375 (malignant melanoma), A549 (epithelial cell lung carcinoma), MIA PaCa-2 (pancreatic carcinoma), and HT29 (colon carcinoma) were used in cytotoxici-ty experiments. Additionally, the human diploid cell line WI-38 (fetal lung epithelial fi-broblasts) was used to assess toxicity in non-malignant human cells. All cells were cul-tured in DMEM (Dulbecco’s Modified Eagle’s Medium, Gibco, Paisley, UK) supplemented with 10% heat-activated fetal bovine serum (FBS, Gibco, Paisley, UK), 2 mM L-glutamine, 1 mM sodium pyruvate, 18 mM HEPES, 26 mM NaHCO_3_, and 50 µg/mL penicillin/streptomycin solution (Gibco, Paisley, UK) and maintained at 37 °C in a 5% CO_2_ atmosphere. 

### 4.4. Hemolytic Activity Assay

Hemolytic activity was evaluated by measurement of hemoglobin release. Fresh human blood was extracted from a healthy donor at the Clinic University of Ulm, Germany, and stabilized with Heparin. The whole blood sample was washed with Dulbecco’s PBS by centrifugation at 3000× *g* for 5 min at 4 °C, and the plasma was discarded. The washing procedure was repeated three times until the supernatant was clear, allowing the isolation hRBC. The remaining pellet was then re-suspended in PBS to obtain a 1% erythrocyte suspension. The peptide Cm-p5 was prepared at an initial concentration of 2048 μM in PBS, added to the first well of a 96-well plate, and diluted serially by a factor of 1/2, resulting a final volume of 100 μL of sample in every well. PBS and 0.1% of Triton X-100 (100 μL) were used as negative and positive controls, respectively. 100 μL of hRBCs suspension was added to each sample in a V-shape 96-well plate and incubated for 2 h at room temperature, with a final range of peptide concentration (1024-1 μM). The plate was centrifuged, 100 μL of the supernatant was carefully transferred to a flat-bottom 96-wells plate, and the absorbance was measured at 540 nm with a microplate reader (Tecan Infinite F200 microplate reader (Tecan Group Ltd., Männedorf, Switzerland). The hemolytic activity was expressed by the hemolysis percentage as the following formula: hemolysis percentage = (OD(test) − OD(negative control))/(OD(positive control) − OD(negative control)) × 100%. The graphs were obtained using GraphPad Prism software (version 8.0) (GraphPad Software, La Jolla, CA, USA).

### 4.5. Cytotoxicity Assays

The cytotoxic activity of Cm-p5 was assessed by determining cell viability with the 3-(4,5-dimethylthiazol-2-yl)-2,5-diphenyltetrazolium bromide assay (MTT, Merck, Darmstadt, Germany). Cells (5 × 10^4^ per well) were distributed in a 96-well plate and incubated overnight at 37 °C to allow adherence. Cells were then treated with Cm-p5 dissolved in serum-free medium at concentrations ranging from 1 to 64 µM for 4 and 24 h. Post-incubation, 20 µL of MTT at 5 mg/mL was applied to each well, and the plates were incubated in the dark for 4 h at 37 °C. The excess of MTT was carefully removed, and 100 µL of dimethyl sulfoxide was added to dissolve formazan crystals. Finally, absorbance was measured at 560 nm and 600 nm with a TECAN infinite M200 microplate reader (Tecan Group Ltd., Männedorf, Switzerland). Cells treated with medium were used as negative control and set as 100% viability. The experiments were performed in triplicate, with the average of three independent experiments reported. IC50 values (concentration at which viability is reduced to 50%) were calculated with a non-linear regression GraphPad Prism software (version 8.0) (Graphpad Software, La Jolla, CA, USA).

### 4.6. Morphological Analysis by Brightfield Microscopy and Double Dapi/Pi Stain

Analysis of morphological changes in A375 cells treated with the Cm-p5 peptide was performed by brightfield microscopy and a double-stain assay with 4′,6-diamidino-2-phenylindole (DAPI) and propidium iodide (PI). For both procedures, cells were seeded on 8-well chamber-coupled slides (Lab Teck Chamber Slides, Costar, Nunc, Denmark) at a concentration of 5 × 10^4^ cells per well in DMEM medium supplemented with 10% FBS and incubated for 24 h at 37 °C. Cells were then treated with various concentrations of Cm-p5 (1 to 64 µM) for 24 h. After incubation, brightfield micrographs of live cells were taken with a Leica DMi8 microscope at 200× magnification (Leica Microsystems CMS GmbH, Wetzlar, Germany). For DAPI/PI staining, three washes with PBS were performed after peptide treatment, and the cells were stained first with DAPI (Sigma-Aldrich, St. Louis, MO, USA) at 300 nM in the dark for 15 min at room temperature. After three washes with PBS to remove the excess DAPI, the cells were incubated with PI (Sigma-Aldrich, St. Louis, MO, USA) at 1 μg/mL in the dark for 15 min. Subsequently, cells were washed three times with PBS and fixed with a 4% paraformaldehyde solution for 10 min. Cells were then washed three times with PBS, and nuclear/cellular morphology as well as membrane permeabilization were analyzed by fluorescence microscopy on a Leica DMi8 fluorescence microscope at 200× and 630× magnification (Leica Microsystems CMS GmbH, Wetzlar, Germany).

### 4.7. Measurement of Cytoplasmic Membrane Depolarization with Disc3 (5)

The effects of the peptide Cm-p5 on the membrane potential of A375 melanoma cells were determined using the membrane potential-sensitive fluorescent dye 3,3′-dipropylthiadicarbocyanine iodide DiSC3 (5) (Sigma-Aldrich, St. Louis, USA). A375 cells (5 × 10^4^ cells/well) were seeded in dark clear-bottomed 96-well plates in DMEM medium supplemented with 10% FBS and incubated overnight at 37 °C in a 5% CO_2_ atmosphere. The medium was removed, and cells were incubated with 100 μL of 2 μM DiSC3 (5) staining solution (dissolved in PBS) for 30 min at 37 °C protected from the light. Then, the cells were washed thrice with PBS and treated with Cm-p5 (1–64 μM) dissolved in PBS. Cells treated with PBS served as a negative control and were measured for 5 min prior to the peptide addition. Fluorescence intensity was monitored (λex = 635, λem = 670 nm) every minute for 25 min in a TECAN infinite M200 microplate reader (Tecan Group Ltd., Männedorf, Switzerland).

### 4.8. Localization of FITC-Labeled Cm-p5

The interaction of Cm-p5 with A375 cells and its cellular localization were determined by double staining with the peptide chemically conjugated with FITC and PI. A375 cells were seeded on slides coupled with 8-well chambers (Lab Teck Chamber Slides, Costar, Nunc, Denmark) at a density of 5 × 10^4^ cells per well in DMEM medium supplemented with 10% FBS and incubated for 24 h at 37 °C in a 5% CO_2_ atmosphere to allow adherence. Subsequently, cells were treated with different concentrations of FITC-labeled peptide for 24 h at 37 °C. After three washes with sterile PBS, 5 µL of PI was added to each well and incubated for 15 min. Samples were then fixed with 4% formaldehyde solution for 10 min. Finally, the wells were washed three times with PBS, and the cells were analyzed using a Leica DMi8 fluorescence microscope (Leica Mycrosystems CMS GmbH, Wetzlar, Germany).

### 4.9. Annexin V FITC/PI Staining for Assessment of Apoptosis/Necrosis

The exposure of phosphatidylserine on the cell surface and the disruption of the cytoplasmic membrane were determined by flow cytometry as indicators of cellular death. Cells were seeded at a density of 1 × 10^5^ cells per well in 24-well plates for 24 h at 37 °C and then treated with Cm-p5 (1 to 64 µM) for 24 h. After three washes with PBS to remove excess peptide, the cells were collected by enzymatic digestion with trypsin (Sigma-Aldrich, St. Louis, MO, USA). Cells were then resuspended in 500 µL of Annexin binding buffer, and 10 µL of Annexin V-FITC (Thermo Fisher, Waltham, MA, USA) was added and incubated for 15 min in the dark at room temperature. Finally, 5 µL of PI was added to each sample and incubated for a further 15 min. At least 1 × 10^4^ cells from each sample were analyzed on a Gallios flow cytometer (Beckam Coulter, Brea, CA, USA). Cells treated with culture medium were used as negative control. 

### 4.10. Cell Cycle Analysis 

DNA content was stained with PI for cell cycle analysis to check whether the alterations in A375 cells treated with Cm-p5 were due to possible cell cycle arrests. The cells were treated with Cm-p5 (1 to 64 µM) for 24 h and collected with trypsin (Sigma-Aldrich, St. Louis, MO, USA). Cells were resuspended in 0.5 mL of PBS and applied to tubes containing 70% cold ethanol, mixing vigorously. The tubes with the cells in ethanol were kept on ice (chamber at 4 °C) for 2 h. After this incubation period, ethanol was removed by centrifugation (200× *g* for 5 min), and cells were washed twice in PBS with 0.5% bovine serum albumin. The staining solution was prepared by adding 2 mg of RNase (Sigma-Aldrich, St. Louis, MO, USA) and 10 µL of PI at 1 mg/mL to 10 mL of 0.1% Triton-X-100 in PBS. Cells were centrifuged again (200 g for 5 min) and resuspended in 1 mL of staining solution. After 30 min of incubation at 37 °C, cells were analyzed on a Gallios flow cytometer (Beckam Coulter, Brea, CA, USA). The percentage of cells in each cell cycle phase was determined. Cells treated with culture medium were used as a negative control.

### 4.11. Analysis of Cell Proliferation by CFSE Assay

To evaluate the ability of cancer cells to recover from the treatment with Cm-p5, the proliferation of the A375 cell line was determined by staining with Carboxyfluoreszin-Succinimidyl-Ester (CFSE) (Thermo Fisher, Waltham, MA, USA). After the treatment of 5 × 10^4^ A375 cells with 1 to 64 µM of Cm-p5 for 24 h at 37 °C, the remaining cells were collected using trypsin (Sigma-Aldrich, St. Louis, MO, USA) and washed three times with PBS, keeping them in 500 µL of PBS supplemented with 2% FBS. Then, 10 µL of CFSE was added to each cell suspension and incubated for 10 min at 37 °C. After three washes with PBS, the cells were seeded again in 96-well plates in 200 µL of cell culture medium supplemented with 10% FBS. The cells were incubated at 37 °C in a 5% CO_2_ atmosphere for 7 days and monitored daily under a microscope. At the end of this period, the cells were collected and analyzed in a Gallios flow cytometer (Beckman Coulter, Brea, CA, USA). Cells treated with culture medium were used as a negative control, and based on the fluorescence intensity given by the presence of CFSE, three main proliferation groups were identified. 

### 4.12. Evaluation of ROS Production

Reactive oxygen species production was measured using the H2DCFDA-Cellular ROS Assay Kit (Hello Bio, Dunshaughlin, Republic of Ireland, HB7375), following the manufacturer’s instructions. Briefly, A375 cells were cultured in the dark, clear-bottomed 96-well plates (5 × 10^4^ cells/well) in DMEM medium supplemented with 10% FBS and incubated for 24 h at 37 °C in a 5% CO_2_ atmosphere to allow adherence. The cells were washed with 1X assay buffer and incubated with 100 µL of 20 µM H2DCFDA working solution for 45 min in the dark at 37 °C to allow dye loading. After this incubation, the cells were washed again with assay buffer and treated with Cm-p5 (1–64 µM) for 24 h at 37 °C. The conversion of non-fluorescent H2DCFDA into the highly fluorescent dichlorofluorescein (DCF) was measured (λex = 485 nm, λem = 535 nm) using a TECAN infinite M200 microplate reader (Tecan Group Ltd., Männedorf, Switzerland). Cells treated with pyocyanin at 1 mM for 2 h and non-treated cells were used as positive and negative controls, respectively.

### 4.13. Caspase Activity Detection

Caspase-3/7 activity in A375 cells treated with Cm-p5 was detected using the Caspase 3/7 Activity Assay Kit (Elabscience, Houston, TA, USA, E-CK-A383, Colorimetric method) following the manufacturer’s protocol. Briefly, A375 cells were seeded in a six-well plate at a density of 2 × 10^5^ cells and incubated overnight to reach confluency. Post-incubation, the cells were treated with different concentrations of Cm-p5 (1–64 µM) for 24 h at 37 °C, and unstimulated cells were used as negative controls. After treatment, the cells were trypsinized and washed with cold PBS for further incubation with lysis buffer for 30 min at 4 °C. Cells were centrifuged at 12,000 rpm for 15 min at 4 °C, and the supernatants containing caspase-3 and 7 were collected. The reaction mixture was prepared by adding the protein sample, reaction buffer, and the substrate Ac-DEVD-pNA at a final volume of 100 μL and incubated at 37 °C for 4 h. Finally, absorbance was measured at 405 nm using a Multiskan microplate spectrophotometer (Thermo Fisher Scientific, Waltham, MA, USA), and the activity of Caspase-3/7 was calculated. The results are representative of three independent experiments performed in triplicate.

### 4.14. Statistical Analysis

All data represent the mean ± standard deviation (SD) of three independent experiments. Differences between the two groups were analyzed using a two-tailed Student’s t-test, and comparisons between multiple groups were performed using analysis of variance (ANOVA) and Dunnett’s test. Compared to the controls, a probability value of *p* < 0.05 was taken as the level for significant differences, * *p* < 0.05, ** *p* < 0.01, and *** *p* < 0.001. Statistical analyses were performed using GraphPad Prism Software version 8.0 (Graphpad Software, La Jolla, CA, USA). Flow cytometry results were analyzed with the software FlowJo (version 10.0, Carrboro, NC, USA). In addition, the software ImageJ (version 1.54, National Institutes of Health, Bethesda, MD, USA) was used to process fluorescent microscopy images. 

## 5. Conclusions

In this study, we demonstrate for the first time the selective cytotoxic effect of the antifungal peptide Cm-p5 on the human melanoma cell line A375. The peptide induces a concentration-dependent membrane depolarization that leads to increased ROS production and loss of membrane integrity. These events culminate in cell death, as evidenced by Annexin V-FITC/PI staining. Interestingly, Cm-p5 also exhibits a long-term antiproliferative effect at low concentrations, highlighting its potential as a dual-action anti-cancer agent. These results reinforce the therapeutic value of HDPs derived from marine invertebrates as promising candidates for the development of alternative or adjunct treatments for aggressive malignancies such as melanoma. Further studies are required to elucidate the specific molecular pathways triggered by Cm-p5 and to assess its in vivo efficacy and immunomodulatory properties. 

## Data Availability

All data generated or analyzed during this study are included in this published article.

## Supplementary Materials

The following are available online at https://www.mdpi.com/article/10.3390/md23070273/s1, Figure S1: (A) RP-HPLC and (B) ESI-MS of crude Cm-p5 peptide. Figure S2: (A) RP-HPLC and (B) ESI-MS of crude N-terminal fluoresceinated Cm-p5.
