# Supplementary material for: The Invertebrate-Derived Antimicrobial Peptide Cm-p5 Induces Cell Death and ROS Production in Melanoma Cells
Source: Mar Drugs. 2025 Jun 29;23(7):273. doi: 10.3390/md23070273 (PMC12299483; doi:10.3390/md23070273)
Supplement: Supplementary file 1 [file marinedrugs-23-00273-s001.zip › marinedrugs-3671825-supplementary.pdf]

marine drugs

Article

The invertebrate-derived antimicrobial peptide Cm-p5 induce cell death and ROS production in melanoma cells

Ernesto M. Martell-Huguet [1,3], Daniel Alpízar-Pedraza [2], Armando Rodriguez [3,4], Marc Zumwinkel [5], Mark Grieshober [5], Fidel Morales-Vicente [6], Ann-Kathrin Kissmann [7], Markus Krämer [7], Steffen Stenger [5], Octavio L. Franco [8], Ludger Ständker [3], Anselmo J. Otero-Gonzalez [1,*] and Frank Rosenau [7,*].

[1] Center for Protein Studies, Faculty of Biology, University of Havana, 25 and I, 10400 La Habana, Cuba; aotero@fbio.uh.cu

[2] Center for Pharmaceutical Research and Development, 26th Avenue, No 1605, Nuevo Vedado , P.O.Box 10400, La Habana, Cuba.

[3] Core Facility for Functional Peptidomics, Ulm Peptide Pharmaceuticals (U-PEP), University Ulm, Faculty of Medicine, Ulm University, 89081 Ulm, Germany; armando.rodriguez-alfonso@uni-ulm.de; ludger.staendker@uni-ulm.de.

[4] Institute of Medical Microbiology and Hygiene, University Clinic of Ulm, Albert-Einstein-Allee 11, Ulm D-89081, Germany; mark.grieshober@uniklinik-ulm.de; barbara.spellerberg@uniklinik-ulm.de; steffen.stenger@uniklinik-ulm.de

[5] Synthetic Peptides Group, Center for Genetic Engineering and Biotechnology, La Habana 10600, Cuba; femvicente@gmail.com

[6] Center for Biochemical and Proteomics Analyses, Catholic University of Brasilia, Brasilia, Brazil.

[7] Institute of Pharmaceutical Biotechnology, Ulm University, Ulm 89081, Germany; ann-kathrin.kissmann@uni-ulm.de; markus-1.kraemer@uni-ulm.de; frank.rosenau@uni-ulm.de

* Correspondence: AO-G; aotero@fbio.uh.cu, (535) 500 5396); FR, frank.rosenau@uni-ulm.de

1. Supplementary Materials

Additional Figures and Tables

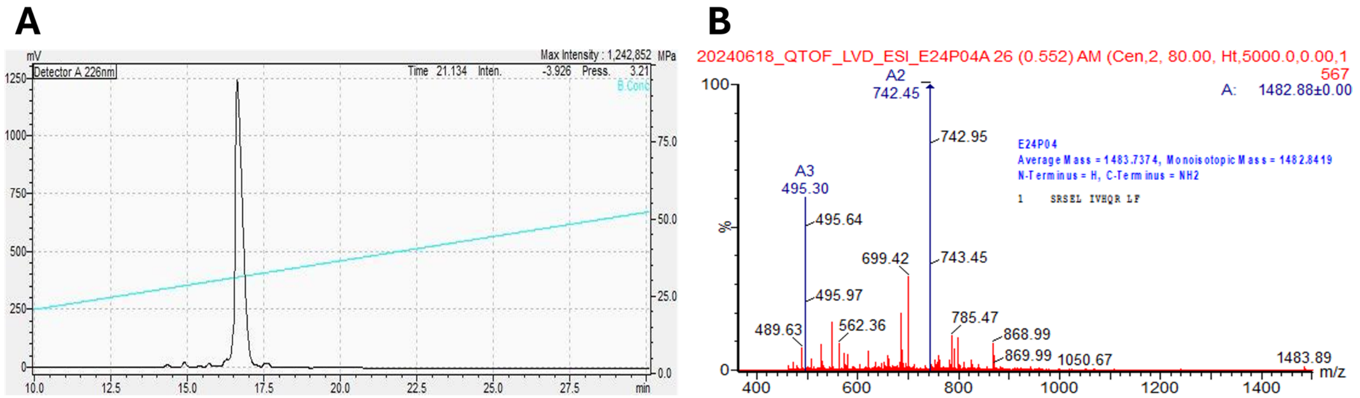

Figure S1: (A) RP-HPLC and (B) ESI-MS of crude Cm-p5 peptide. The peptide was obtained with more than 95% purity.

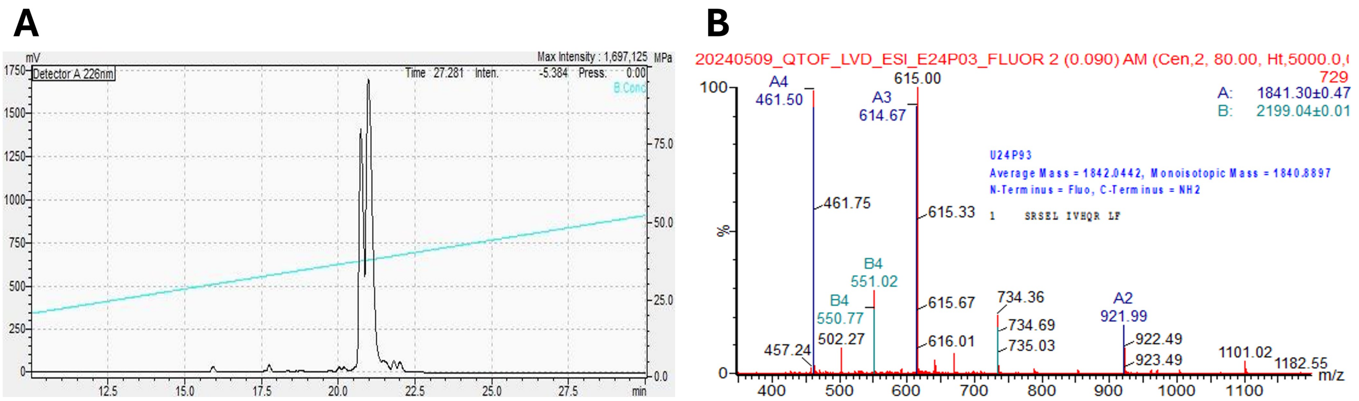

Figure S2: (A) RP-HPLC and (B) ESI-MS of crude N-terminal fluoresceinated Cm-p5. The peptide was obtained with more than 95% purity.

Table S1. IC50 (µM) of Cm-p5 in different cancer cell lines at 4 and 24 hours of treatment.

Cell Lines	IC 50 4h	IC 50 24h
A375	68.89 ± 1.10	1.58 ± 1.25
HT-29	37.07 ± 1.26	20.24 ± 1.63
MIA PaCa-2	>64	>64
A549	>64	>64

IC_{50} (half-maximal inhibitory concentration) of Cm-p5 at 4 and 24h in each cancer cell line. IC50 values and that exceeded the concentrations of Cm-p5 evaluated in the assays are represented as >64 µM. A375 (malignant melanoma), HT-29 (colon carcinoma), MIA PaCa-2 (pancreatic carcinoma), and A549 (epithelial cell lung carcinoma).

Disclaimer/Publisher's Note: The statements, opinions and data contained in all publications are solely those of the individual author(s) and contributor(s) and not of MDPI and/or the editor(s). MDPI and/or the editor(s) disclaim responsibility for any injury to people or property resulting from any ideas, methods, instructions or products referred to in the content.
